# 

**Published:** 1864-10

**Authors:** 


BOOKS RECEIVED.

*A Comprehensive Medical Dictionary, containing the Pronunciation, Etymology and Signification of the terms made use of in Medicine and the kindred sciences, with an Appendix, comprising a complete list of all the more important articles of the Materia Medica, arranged according to their medicinal properties; also an explanation of the Latin terms and phrases occurring in Anatomy, Pharmacy, etc.; together with the necessary directions for writing Latin prescriptions, etc., etc.* By J. Thomas, M. D. *Philadelphia:* J. B. Lippincott & Co., 1864.

*A Statement of the causes which led to the dismissal of Surgeon-General Wm. A. Hammond from the Army; with a review of the evidence adduced before the Court.*

*Lectures on Venereal Diseases, by* Wm. A. Hammond, M. D. *Philadelphia:* J. B. Lippincott & Co., 1864.

